# A Contribution to the History of Japanese Education Systems for Radiological Technologists

**DOI:** 10.14789/jmj.JMJ21-0028-R

**Published:** 2021-12-17

**Authors:** YASUAKI SAKANO, KENZO MUROI, MASAMI GOTO, HAJIME SAKAMOTO, YUH MORIMOTO, SHINSUKE KYOGOKU, TATSUO SAKAI, HIROYUKI DAIDA

**Affiliations:** 1 Department of Radiological Technology, Juntendo University Faculty of Health Science, Tokyo, Japan; 1 Department of Radiological Technology, Juntendo University Faculty of Health Science, Tokyo, Japan; 2 Juntendo University Faculty of Health Science, Tokyo, Japan; 2 Juntendo University Faculty of Health Science, Tokyo, Japan

**Keywords:** radiological technologist, education system, history, globalization

## Abstract

**Background:**

The evolution of radiological technology is one of the most remarkable events of modern medical technology. Radiological examination has resulted in non-invasive, individual diagnostic imaging, which has contributed significantly to successful medical treatment of patients.

**Key Concepts:**

This review summarizes past and current Japanese educational systems for radiological technologists with a historical perspective focusing on three periods. The first period begins with Roentgen's discovery of X-rays (1895), the second period begins with the establishment of the Radiological X-ray Technologist Act (1951), and the third period begins with the launch of the first university course for radiological technologists (1987). It is conceivable that those periods are in accordance with the technological paradigm shifts, including the development of contrast radiography and the application of CT and MRI to clinical practice. To maintain awareness of the most recent available technologies and maximize safety, educational programs teaching the latest knowledge were offered during each period.

**Conclusions:**

The advanced technologies require highly skilled radiological technologists and highly established educational systems. At present, over 70% of Japanese educational programs for radiological technologists are university courses leading to a bachelor's degree. The increasing globalization of radiological technology requires future radiological education systems to have a global perspective.

## Introduction

Radiological technology is essential for high-quality modern healthcare and is used at present not only for diagnostic imaging but for radiation therapy and nuclear medicine. X-ray examinations, introduced at the end of the 19th century, were the first type of non-invasive diagnostic imaging applied to clinical settings^1)^. Before the advent of X-ray technology, the inside of the human body had been visualized for diagnosis of diseases by cadaveric autopsy only after death^1)^. Incorporation of X-ray examination into clinical practice allowing in vivo visualization of the human body contributed significantly to successful treatment^2)^.

Radiological examinations were initially applied for morphological diagnoses of, for example, orthopedic patients and patients with tuberculosis^2)^. The safety and quality of contrast media have been improved, leading to advances in the field of contrast radiography. Later, two remarkable cross-sectional diagnostic imaging technologies, computed tomography (CT) and magnetic resonance imaging (MRI), were developed in the 1970s to 1980s. Radiological examinations have been gradually substituted for the autopsy as the diagnostic means.

Radiological technology is highly developed in Japan, with patients frequently undergoing diagnostic radiological examinations. According to the Organization for Economic Co-operation and Development (OECD), the numbers of MRI and CT units per person in Japan are the highest among all countries surveyed^3)^. The advanced technologies require competent, skilled radiological technologists (RTs). Education systems updated to fit technological advances are crucial to train skilled RTs and to maintain their knowledge of the latest clinical practices. The transition of the education system is, therefore, a reflection of the development of radiological technology.

This review summarizes past and current Japanese education systems for RTs with a historical perspective focusing on three periods. Based on the educational and technical development, we determined that the first period begins with Roentgen’s discovery of X-rays (1895), the second period begins with the establishment of the Radiological X-ray Technologist Act (1951), and the third period begins with the launch of the first four-year university course for radiological technologists (1987). During the first period, Shimadzu X-Ray Technology Training Center (the current Kyoto College of Medical Science), the first X-ray technical education institution in Japan, was established. In the beginning of the second period, Radiological X-ray Technologist Act and X-ray technologist training school designation rule were enacted. In this period, Chuoh College of Medical Technology, the oldest training institution in Tokyo was established in 1959. Since the third period, technological development of radiological technology has been accelerated. Today, there are three types of training schools, universities, professional training colleges, and a training institution run by Japan Self-Defense Forces, comprising a total number of 55 institutions. The universities are accredited by Ministry of Education, Culture, Sports, Science and Technology (MEXT), and all courses are standardized by Ministry of Health, Labour and Welfare (MHLW). The Domestic Radiological Technologist Education Facility Council that oversees those 55 institutions contributes to standardization and improving the education level of RTs.

The present review attempted to reveal historical events in the education system of radiological technology, using several re-discovered references. The article also discusses future perspectives of Japanese radiological technology and RTs. Literature on the Japanese history of radiological technology and publications discussing the educational systems are scarce, and to our best knowledge, this is the first English article to describe the Japanese education system for RTs within a historical context.

## 1) Beginning of 20th century: Including Roentgen’s discovery of X-rays (1895)

A few months after the discovery of X-rays by Wilhelm Conrad Röntgen at the end of 1895, two Japanese groups successfully produced radiographic photographs^4)^, with one of the first radiographic photographs, showing Japanese swords, published in a journal in April 1896^5)^. Initially, medical use of X-rays in Japan occurred in the military^6)^. Dr. Eijiro Haga, an army surgeon at that time, reported using X-ray examinations in 1901 in the treatment of soldiers wounded during the Boxer Rebellion^7)^. X-ray equipment used at field hospitals was highly regarded during the Russo- Japanese War (1904-05)^4, 8)^. The early technologies used in radiography were described in an article published in 1906^8)^. By using a 110 V DC 70 cm inductor, X-ray images of the limbs required 3 to 4 minutes and images of the chest, thigh, and head required 10 minutes8. Changing the photographic plates significantly shortened the exposure time, to 5 to 30 seconds for the limbs and 15 to 60 seconds for the chest, spine, and thighs^8)^.

**Figure 1:**
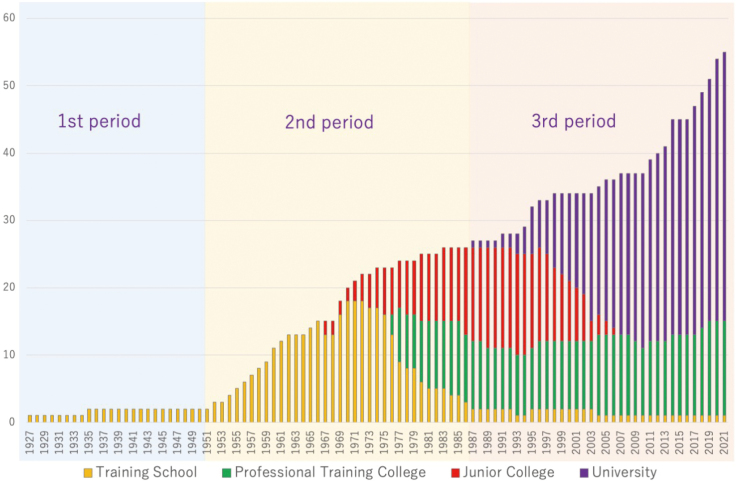
Transition in the education systems in radiological technologists from 1927 to 2021 The number of institutions is retrieved according to the institutions that are currently active. There may have been institutions that existed in the past but were not included because of the lack of records.

In the early 1910s, X-ray radiology was taught at several military medical schools^6)^. Knowledge and methods of radiology were also taught in apprenticeships at universities, hospitals, and clinics^6)^. The importance of education about radiological technology was well recognized by the 1920s. The first two publicly available education programs for radiological technology in Japan were organized by Tokyo Denki (the current Canon Medical Systems Corporation) in 1918 and Shimadzu Corporation in 1921^6, 9)^. The contents of the latter can be assumed from X-ray lecture records published by Shimadzu corporation in 1923. They comprise nine volumes by various authors among whom most held academic titles in medicine, engineering, and science^10)^. Among 86 articles in the volumes, the authors of 59 were medical doctors who contributed topics related to clinical practice. Among the other articles are topics including introduction of X-rays and X-ray tubes, physics, electricity, and X-ray generators^10)^. Shimadzu Corporation, a manufacturer of precision, measuring, and medical instruments, produced the first medical X-ray equipment in Japan^9)^. A seminar organized by Shimadzu Corporation was the foundation of Shimadzu X-Ray Technology Training Center, the first X-ray technical education institution in Japan (established, 1927). It is believed that the number of X-ray technologists in Japan was 500 to 700 by the end of the Taisho era (1912-1926)^11)^. As demonstrated in Figure 1, the number of training institutions known today is few in this period. This indicates that many technologists were trained in various different programs, which were not standardized by the national authority.

## 2) Mid-20^th^ century: Including establishment of the Radiological X-ray Technologist Act (1951)

The increases in the numbers of radiological examinations during the middle of the 20^th^ century resulted in the profession of RT becoming widely recognized and established in Japan. Following several difficult years required for reconstruction after World War II, the establishment of the profession of RT began in the 1950s. The Radiological X-ray Technologist Act was enacted in 1951, and a two-year educational program accredited and standardized by MHLW was started the next year^12)^. The first national examination for X-ray technologists took place in 1954. Prior to that, the first national examination designed for accreditation of those working as medical X-ray technologists was held in 1952. Because of increases in radiological applications in medicine, the Radiological X-ray Technologists Act was renewed as the Radiological Technologist Act in 1968^13)^, with the first national examination for RTs taking place the same year and continuing since then. The education program was gradually extended from two to three-years in Junior College (Table 1, Figure 1).

**Table 1:** Historical milestones of the Japanese education system for radiological technologists

Year	Event
1918	Start of an educational program for radiographers by Tokyo Denki
1921	Start of an educational program for radiographers by the Shimadzu Corporation
1927	Establishment of the first X-ray technical education institution in Japan
1951	Enactment of the X-ray Technologist Act
1952	Start of a 2-year educational program accredited by the MHLW First national examination for non-certified X-ray technologists
1954	First national examination for X-ray technologists (terminated in 1984)
1956	Termination of the national examination for non-certified X-ray technologists
1968	Enactment of the Radiological Technologists Act First national examination for Radiological Technologists
1987	Establishment of the first bachelor's program for Radiological Technologists by Fujita Health University

During this period, a remarkable event in radiological technology was the emergence of contrast radiography. Egas Moniz, a Portuguese neurosurgeon, discovered contrast angiography in 1927, enabling the imaging of cerebral blood vessels and vascular alterations as well as other intracranial disorders^14)^. Two years later, Reynaldo dos Santos, another Portuguese physician, introduced translumbar aortography^15)^. Following further developments in angiography, it began to be used in clinical applications. An innovative method using a catheter to gain vascular access, was introduced in 1953. This method, called the Seldinger technique, enabled angiography to be performed safely^16)^. Angiography has since been applied to excretory urography, the thoracic aorta, coronary vessels, the renal artery, and the diagnosis of aortic aneurysms^15, 17, 18)^. Interventional radiography (IR), first introduced in 1967, is a low-invasive procedure widely used for both diagnostic and treatment purposes^19)^. IR initially involved the use of X-ray fluoroscopy, but methodological advances have enabled IR to be performed using a combination of CT and ultrasound^20)^.

Improvements in the safety and quality of contrast media are associated with the evolution of contrast radiography^15)^. In the 1950s and 1960s, iodine-based contrast media were used, causing many side effects, including pain, dizziness, and occasional death^21)^. Non-ionic contrast media were introduced in the 1970s, initially applied to myelography^21)^. As alternatives to these hyperosmolar contrast media, low-osmolar contrast media were developed in the 1980s, significantly reducing the incidence of side effects^21)^.

Another historically important clinical application of contrast radiography is double-contrast radiography using barium^22)^. In Japan, routine examinations for the early detection of gastric cancer using an X-ray fluoroscopy were introduced in the 1950s^23, 24)^, with nationwide examinations continuing for decades^25)^. At present, routine screening for early gastric diseases involves double- contrast radiographic examinations combined with gastrointestinal tract examinations by endoscopy^24)^.

## 3) Late 20^th^ century to date: Including the launch of university courses for radiological technologists (1987)

The introduction of CT and MRI into clinical practice in the late 20^th^ century was one of the biggest paradigm shifts in radiological examinations. X-ray CT was introduced by an English electrical engineer, Godfrey Hounsfield, who received a Nobel Prize in 1979 for the development of this remarkable technology^26)^. To accrue data, an X-ray tube and a detector are arrayed on the opposite sides of a circle, which spins around the patient’s body^26)^. In Japan, the first CT was installed in Tokyo Women’s Medical University Hospital in 1975^27)^. The initial CT machines were only for the head, with each scan taking 4 minutes^28)^. The scanning method evolved to shorten the time required to scan the entire body. Shifting from a pencil beam to a narrow fan beam shortened the scanning time to 20 seconds, after which the technique was altered from translate/rotate to rotate/rotate methods^28)^. In the late 1980s, a helical scan was introduced, which moves the bed while rotating the X-ray tube and detector array^28, 29)^. Detector arrays improved from single to multiple detectors placed in collimation, enabling the scanning of a wide range of the body with many cross-section images in each rotation^29)^. At present, a scan from the chest to the pelvis takes a few seconds, and the use of CT over the last few decades has become widespread. The combination of CT or MRI with positron emission tomography (PET), has led to the widespread use of PET-CT and PET-MRI examinations since the 1990s. Conformation radiotherapy that requires the three-dimensional information of the irradiation site rapidly emerged in the early 1980s, upon the clinical application of CT^30)^. The development of a CT apparatus specialized for the treatment planning system launched in 1984, with clinical application began in 1987^30)^.

**Figure 2:**
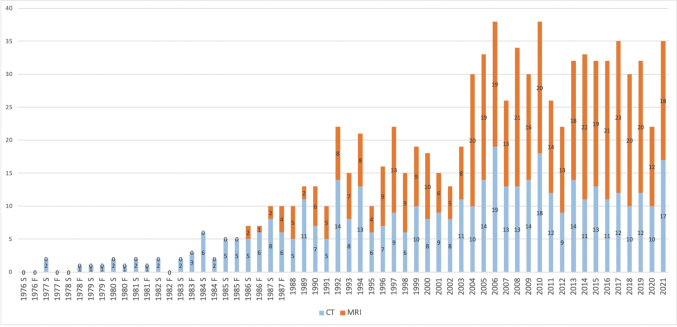
Number of questions related to CT or MRI on national examinations (1976 to date) Until 1987, the examination was conducted twice a year. S: spring examination; F: fall examination. Data from 1976 to 1977, from 1978 to 1982, from 1983 to 1985, from 1986 to 1989, from 1990 to 1994, and from 1995 to 2000 were obtained from the 1979, 1984, 1987, 1990, and 2000 editions, respectively, of the National Examination Questions (Kanehara shuppan). Data from 2001 to the present were obtained from National Examination Questions (Iryo kagaku sha)

The impact of new technology on the curriculum of educational programs in RT can be observed by evaluating trends in the national examination. Figure 2 illustrates the number of questions related to CT and MRI in the national examination from 1976 to 2021. Questions related to CT and MRI emerged in the spring of 1977 and the spring of 1986, respectively, with the number of questions gradually increasing since then. From 2011 to 2021, the average numbers of questions per year related to CT and MRI were 13.1 and 20, respectively. In the past decade, the questions related to CT or MRI constituted approximately 17% of the 200 questions on the national examination.

In parallel with advances in these technologies, the role of RTs has expanded, with a wider range of subjects included in educational programs. This led to the introduction of university courses for RTs, the first offered by Fujita Health University in 1987, followed by Suzuka University of Medical Science in 1991 and Osaka University in 1993^31)^. The transition to the university courses in this period is shown in Figure 1.

As of 2021, 55 institutions provide educational programs for RTs in Japan, with 40 (73%) of these institutions providing university level courses, and 15 (27%) providing 3-year courses^32, 33)^. Of these 15 institutions, 14 are professional training colleges, and one is Institute of Medical Radiology Technologists run by Japan Self-Defense Force (JSDF) (Table 2)^33)^. Universities offer bachelor’s degrees, and professional training colleges offer diplomas equivalent to associate degrees^34)^. National examinations are held once a year and are open to anyone who has completed a program at a university, professional training college, or JSDF. Some professional training colleges offer nighttime programs, enabling students to work during the day. Moreover, some students have already obtained a bachelor’s degree by the time they enroll in a professional training college. A few professional training colleges will probably remain in the future as they meet the needs of some students.

**Table 2:** Number of educational institutions for radiological technologists (as of 2021)

	Course duration (yrs)	Numbers of institutions by type				
		National	Public	Private	Other	Total
University	4	11	3	26	0	40
Professional training college	3	0	0	14	0	14
Japan Self-Defense Forces	3	0	0	0	1	1

The curricula of all institutions are standardized and regulated by MHLW^35)^, and moreover, universities are accredited by MEXT. Of 47 prefectures in Japan, 26 have educational institutions offering these programs, with Tokyo having the most, 10, including five universities; four professional training college, and the JSDF training institution. In addition, Osaka has six institutions, including three universities and three professional training colleges; Hokkaido has four (three and one, respectively), Fukuoka has four (three and one, respectively), and Aichi has three (two and one, respectively).

## Future perspectives

**Figure 3:**
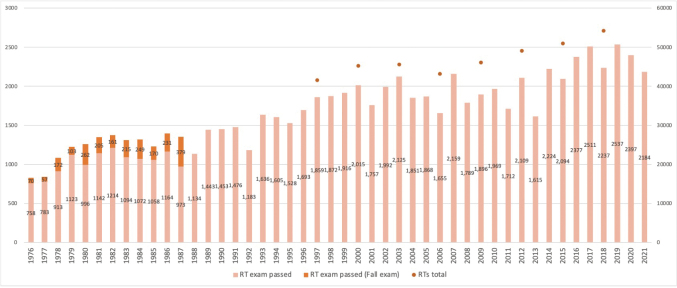
Numbers of RT candidates who passed the national examinations and total numbers of RTs in the workforce by year (1976 to date) Until 1987, the examination was conducted twice a year (spring and fall). Data from 1976 to 1977, from 1978 to 1982, from 1983 to 1985, from 1986 to 1988 were obtained from 1979, 1984, 1987 and 1990 editions, respectively, of the National Examination Questions (Kanehara shuppan). Data from 1989 to the present were obtained from the MHLW website (https://www.mhlw.go.jp/file/05-Shingikai-10801000-Iseikyoku-Soumuka/0000200803.pdf)

Medical services must be of high quality, accessible, and affordable. Simultaneous attainment of these three conditions is barely accomplished in Japan with the contribution of the Japanese healthcare system, in which almost 100% of the population is insured^36)^. The World Health Organization (WHO) has stated that, in general, Japanese hospitals are well equipped with high-technology medical devices, including CT and MRI scanners, and that the costs of these examinations are relatively low^36)^. In addition, patients in Japan are not restricted by any gate-keeping system, allowing them to choose any hospital or clinic^36)^. Consequently, many Japanese patients, even those with mild symptoms, often go to secondary healthcare facilities that have advanced equipment^36)^. Increased attendance at these facilities would therefore require an increase in the number of RTs. The MHLW reported that, in 2017, the number of RTs in the workforce was 54,213, or 1.7% of the total number of healthcare workers, 3,124,321. Over the last 30 years, the number of RTs has increased by 1500 to 2500 each year (Figure 3), with the number predicted to increase to 9.5% in 40 years^37)^.

Aging of the population and emergence of new infectious diseases such as COVID-19 are expected to accelerate developments in radiological technology. The Japanese education system for RTs has evolved in accordance with the needs of Japanese society. However, the increased globalization of healthcare settings will require that the education system attain a global perspective. For example, new digital technologies, such as artificial intelligence (AI) and machine learning, in diagnostic imaging are being intensively studied and becoming competitive. International research collaboration is essential for the development of these emerging technologies^38)^. Future university educational programs should therefore foster RTs with a global mindset.

## Conclusion

The Japanese education system for RTs has evolved in accordance with technological developments. To maintain awareness of the most recent available technologies and maximize safety, educational programs teaching the latest knowledge were offered during each period. At present, over 70% of Japanese educational programs for RTs are university courses leading to a bachelor’s degree. The increasing globalization of radiological technology requires future radiological education systems to have a global perspective.

## Funding

This research did not receive a grant from funding agencies in the public, commercial, or not- for-profit sectors.

## Author contributions

YS devised the project and the main conceptual ideas. KM and MG collected and analyzed the data regarding the radiologist education. HS and SK proved and interpreted the data. YM performed reference investigations and wrote the first draft. TS provided the data regarding the radiologist schools and refined the manuscript. HD supervised the project. All authors provided critical feedback and helped shape the research. All authors read and approved the final manuscript.

## Conflict of interest statement

The authors declare that they have no conflicts of interest regarding this review.
